# Familial mixed nephrocalcinosis as a cause of chronic kidney failure: two case reports

**DOI:** 10.1186/1752-1947-8-355

**Published:** 2014-10-27

**Authors:** Pedro Francisco Ferraz de Arruda, Márcio Gatti, José Germano Ferraz de Arruda, Fernando Nestor Fácio, Luis Cesar Fava Spessoto, Laísa Ferraz de Arruda, José Maria Pereira de Godoy, Moacir Fernandes Godoy

**Affiliations:** 1 Urology Department, São José do Rio Preto School of Medicine, Avenida Brigadeiro Faria Lima 5416, São José do Rio Preto, SP 15090-000, Brazil; 2 Medical Department, União das Faculdades dos Grandes Lagos, (Unilago), Rua Dr. Eduardo Nielsem 960, São José do Rio Preto, SP 15030-070, Brazil; 3 Cardiology and Cardiovascular Surgery Department, São José do Rio Preto School of Medicine, Avenida Brigadeiro Faria Lima 5416, São José do Rio Preto, SP 15090-000, Brazil

**Keywords:** Chronic kidney disease, Kidney, Nephrocalcinosis

## Abstract

**Introduction:**

Nephrocalcinosis consists of the deposition of calcium salts in the renal parenchyma and is considered the mixed form when it involves the renal cortex and medulla. The main etiological agents of this condition are primary hyperparathyroidism, renal tubular acidosis, medullary sponge kidney, hyperoxaluria and taking certain drugs. These factors can lead to hypercalcemia and/or hypercalciuria, which can give rise to nephrocalcinosis.

**Case presentations:**

Patient 1 was a 48-year-old Caucasian woman with a history of bilateral nephrocalcinosis causing chronic kidney failure. Imaging examinations (X-ray, ultrasound and computed tomography of the abdomen) revealed extensive calcium deposits in the renal parenchyma, indicating nephrocalcinosis as the causal factor of the disease. Patient 2 is the 45-year-old brother of patient 1. He exhibited an advanced stage of chronic kidney failure. As nephrocalcinosis is considered to have a genetic component, a family investigation revealed this condition in patient 2.

**Conclusion:**

Nephrocalcinosis may be detected incidentally through diagnostic imaging studies. Whenever possible, treatment should include the base disease that caused the appearance of the calcification, as the precise etiological determination is extremely important.

## Introduction

Nephrocalcinosis is a generalized increase in the concentration of calcium and the deposition of calcium salts in the renal parenchyma [1]. The main etiological agents of this condition are primary hyperparathyroidism, renal tubular acidosis, medullary sponge kidney, idiopathic hypercalciuria, hyperoxaluria (primary, enteric or toxic), secondary hypercalcemia (sarcoidosis, neoplasm and osteoporosis) and drugs (including furosemide, acetazolamide and amphotericin B) [2].

This condition has no clinical symptoms in the early phase, except in cases of nephrolithiasis (kidney stone) and ureteral colic. Polyuria and thirst may be the first symptoms due to the concentrating defect in the renal tubules. The condition can progress to chronic kidney failure, and the prognosis depends on the underlying cause. Laboratory examinations may reveal erythrocytosis, and microscopic pyuria in the urinary sediment is nearly a constant, even in the absence of urinary infection, causing a chronic inflammatory response stemming from calcification [2,3].

Nephrocalcinosis can be detected by diagnostic imaging examinations (radiography, ultrasound and computed tomography) [1,4] which reveal calcium deposits in the renal parenchyma suggesting irreversible lesions accompanied by a variable degree of compromised kidney function [5]. The literature on familial mixed nephrocalcinosis is scarce.

In this report, we describe two cases of familial mixed nephrocalcinosis with progression to chronic kidney failure, followed by dialysis, which allowed for early diagnosis and treatment.

## Case presentations

### Patient 1

**Figure 1:**
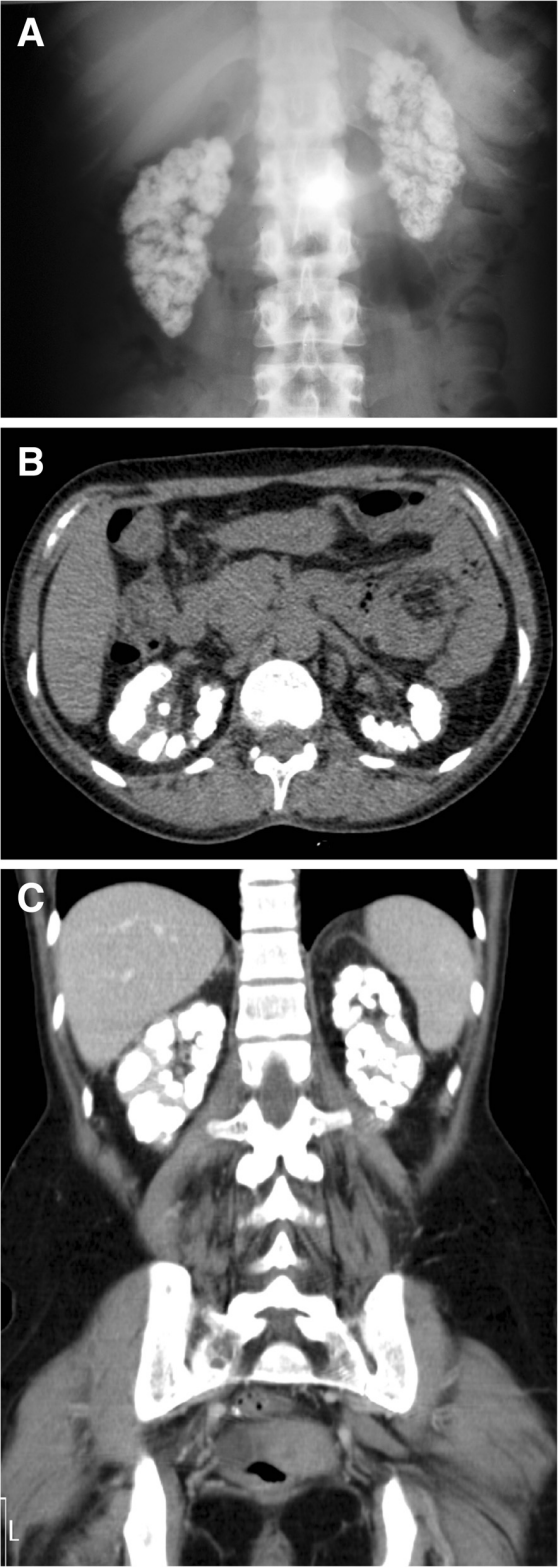
**Calcifications visible in the abdomen of patient 1.** Images reveal mixed nephrocalcinosis with accentuated calcifications in the renal cortex and medulla. **(A)** X-ray. **(B)** Computed tomography (longitudinal cut). **(C)** Computed tomography (coronal cut).

A 48-year-old Caucasian woman with a history of bilateral nephrocalcinosis causing chronic kidney failure presented with co-morbidities such as systemic arterial hypertension and hyperparathyroidism. Imaging examinations (X-ray, ultrasound and computed tomography of the abdomen) revealed extensive calcium deposits in the renal parenchyma, indicating nephrocalcinosis as the causal factor of the disease (Figure 1).

With the progression of her kidney disease, dialysis was needed for approximately two years, and then the patient received a kidney transplant from a deceased donor. The surgery was successful, and her progress was satisfactory during the first year of follow-up. She currently has good general health and adequate kidney function.

### Patient 2

As nephrocalcinosis is considered to have a genetic component [4], a family investigation revealed this condition in the 45-year-old brother of patient 1. He exhibited systemic arterial hypertension, intermittent atrial arrhythmia, left ventricular dysfunction on an echocardiogram and myocardial scintigraphy suggestive of ischemia. The imaging examinations (X-ray, ultrasound and computed tomography of the abdomen) revealed extensive calcium deposits in the renal parenchyma, indicating nephrocalcinosis. He was in an advanced stage of chronic kidney disease (tubulointerstitial nephritis), for which dialysis was initiated. He is currently awaiting a kidney transplant.

## Discussion

Nephrocalcinosis consists of the deposition of calcium salts in the renal parenchyma. The mixed form involves the renal cortex and medulla. This condition differs from kidney stones by the formation of calculi in the excretory tract. The increase in calcium content in the kidneys occurs in three phases (chemical, microscopic and macroscopic) with different degrees of kidney damage. In the present case series, both siblings were in the macroscopic phase, as the calcium deposits had reached dimensions that were visible using diagnostic imaging methods [2,6].

Macroscopic nephrocalcinosis is classified as cortical (3% of cases), medullary (97% to 98% of cases) or mixed (involving the renal cortex and medulla) [2]. The cases described herein are rare, as both patients exhibited familial macroscopic mixed nephrocalcinosis, with no specific symptoms beyond concomitant kidney stones. The mixed variant is a very rare condition and may be seen in primary oxalosis or atypical infection by *Mycobacterium avium-intracellulare* in patients with AIDS [2].

Childhood nephrocalcinosis occurs in the medullary form in most cases and can progress to chronic kidney failure with a need for dialysis during childhood or early adulthood [7]. Although the mixed nephrocalcinosis was diagnosed in the macroscopic phase in the two patients described in this report, the chemical and microscopic phases may have occurred in childhood.

Nephrocalcinosis can be detected using imaging examinations such as radiography, ultrasound or computed tomography [2,3]. Axial computed tomography without contrast is considered adequate for differentiating cortical and medullary nephrocalcinosis and demonstrating the relationship between calcifications in the parenchyma and the calyceal system, allowing the precise localization of the calcifications [5]. In the presently reported cases, the imaging examinations confirmed that both patients were in an advanced stage of nephrocalcinosis.

With kidney stones, patients are encouraged to ingest liquids to obtain a minimum urine production of 1.5L and to make dietary changes such as restricting animal protein consumption and reducing sodium intake to <100mEq/day. Measures are also taken to reduce calciuria and increase inhibitors of urinary crystallization [3-5]. However, these measures were not possible in the two cases we report, owing to the advanced degree of kidney impairment. Whenever possible, the choice of treatment should include the base disease that caused the appearance of calcifications, for which a precise etiological diagnosis is extremely important.

## Conclusion

As nephrocalcinosis can lead to chronic kidney failure and the need for dialysis or a kidney transplant, urologists and nephrologists should be capable of identifying this condition and perform treatment founded on the base disease to avoid or slow down the progress of an unfavorable outcome.

## Consent

Written informed consent was obtained from the patients for publication of this case report and any accompanying images. A copy of the written consent is available for review by the Editor-in-Chief of this journal.

## Competing interests

The authors declare that they have no competing interests.

## Authors’ contributions

PFFA contributed to the conception and design of the report; acquisition, analysis and interpretation of the data; and drafting of the final manuscript. MG contributed to the acquisition and analysis of the data. JGFA contributed to the interpretation of the data and drafting of the final manuscript. FNF and LCFS contributed to the analysis and interpretation of the data. LFA contributed to the analysis and interpretation of the data and drafting of the final manuscript. JMPG contributed to the acquisition and analysis of the data and drafting of the final manuscript. MFG contributed to the conception and design of the report, the analysis and interpretation of the data, and drafting of the final manuscript. All authors read and approved the final manuscript.
